# *PTEN/MMAC1* expression in melanoma resection specimens

**DOI:** 10.1038/sj.bjc.6600653

**Published:** 2002-12-02

**Authors:** M Deichmann, M Thome, A Benner, U Egner, W Hartschuh, H Näher

**Affiliations:** Department of Dermatology, University Clinics of Heidelberg, Vossstraße 2, 69115 Heidelberg, Germany; Central Unit Biostatistics, German Cancer Research Center R0700, Im Neuenheimer Feld 280, 69120 Heidelberg, Germany

**Keywords:** melanoma, *PTEN/MMAC1*, tumour suppressor gene

## Abstract

*PTEN/MMAC1*, a tumour suppressor gene located on chromosome 10q23.3, has been found to be deleted in several types of human malignancies. As the chromosomal region 10q22-qter commonly is affected by losses in melanomas, we addressed this gene as tumour suppressor candidate in melanomas. Investigating *PTEN/MMAC1* expression at mRNA level by semi-quantitative reverse transcription-polymerase chain reaction, we did not find a statistically significant down-regulation in melanoma resection specimens in comparison to acquired melanocytic nevi from which melanomas quite often are known to arise. Upon immunohistochemistry, PTEN/MMAC1 protein expression in melanomas was not lost. Sequencing the *PTEN/MMAC1* cDNAs in 26 melanoma resection specimens (21 primary melanomas, five metastases), we detected three point mutations and two nucleotide deletions which did not represent genetic polymorphisms. With respect to the predicted protein sequences, all three point mutations were silent whereas the two frame shifts at the extreme C-terminus resulted in a loss of the putative PDZ-targeting consensus sequence. As loss of this motif possibly impairs localization and function of PTEN/MMAC1 in the two corresponding primary tumours, alterations of this tumour suppressor protein may participate in some melanomas.

*British Journal of Cancer* (2002) **87**, 1431–1436. doi:10.1038/sj.bjc.6600653 www.bjcancer.com

© 2002 Cancer Research UK

## 

Melanomas are one of the most aggressive of the skin cancers and have shown a dramatic increase in incidence over the past decades (Rigel *et al*, 1996). The genetic principles that underly the development and progression of these tumours are only beginning to emerge. Genomic regions that exhibit losses of heterozygosity (LOH) and therefore suggest the presence of tumour suppressor genes in sporadic melanomas, have been identified, among others, at chromosomes 6q22-27 (Albino *et al*, 1994) and 11q22-23 (Robertson *et al*, 1999). Moreover, LOHs at chromosome 10 occur in 30–60% of both early- and advanced-stage tumours (Newton, 1994; Bastian *et al*, 1998) and are an indicator of a poor clinical prognosis (Healy *et al*, 1998). Segmental deletions were cytogenetically localized to 10q (Richmond *et al*, 1986; Parmiter *et al*, 1988; Isshiki *et al*, 1993; Indsto *et al*, 1998) and subsequently narrowed by studies of LOH to 10q22-qter (Isshiki *et al*, 1993; Herbst *et al*, 1994; Walker *et al*, 1995; Healy *et al*, 1996). This region is also a common target of LOH in a wide spectrum of sporadic cancers, including neoplasias of the prostate (Trybus *et al*, 1996), thyroid (Nelen *et al*, 1996), kidney (Speicher *et al*, 1994), endometrium (Nagase *et al*, 1996), and bone (Raskind *et al*, 1996). Investigating kinetics of melanoma tumour formation in animals, transfer of chromosome 10q distal to 10q23.1 into melanoma cells caused a severe reduction in tumour growth (Robertson *et al*, 1999).

One notable gene at the chromosomal locus 10q23.3 is the tumour suppressor gene ‘*phosphate and tensin hommolog deleted on chromosome ten*’ (*PTEN;* Li *et al*, 1997; Steck *et al*, 1997), also known as ‘*mutated in multiple advanced cancers*’ *(MMAC1)*. By dephosphorylating the phosphatidylinositol-3,4,5-triphosphate, this tumour suppressor negatively controls the phosphoinositide-3-kinase signalling pathway for regulation of cell proliferation and cell survival (Bonneau and Longy, 2000). Deletions and mutations of *PTEN/MMAC1* have been reported in a wide variety of human cancers, including tumours of the brain, the lung, the prostate and the breast (Teng *et al*, 1997; Bonneau and Longy, 2000; di Cristofano and Pandolfino, 2000), placing this gene among the most commonly mutated genes in human cancer. In melanoma cell lines, inactivating deletions or mutations of *PTEN* have been reported in 29–43% (Guldberg *et al*, 1997; Tsao *et al*, 1998, 2000). We therefore looked for *PTEN/MMAC1* alterations in melanoma resection specimens at mRNA and protein level by semi-quantitative RT–PCR, sequencing of cDNA transcripts and immunohistochemistry.

## MATERIALS AND METHODS

### Purification of mRNA from melanoma and nevus resection specimens

Tissue specimens of primary and metastatic melanomas and acquired melanocytic nevi were frozen in liquid nitrogen immediately after resection. Tissues were microdissected to separate tumour from normal tissues. From patients where the *PTEN/MMAC1* cDNAs were found to be altered in the melanomas, blood mononuclear cells were purified by Lymphoprep (Nycomed Pharma, Oslo, Norway) and investigated as non-neoplastic tissues. Total RNA was isolated by anion exchange on resin columns (RNA mini kit, QIAGEN, Valencia, CA, USA), concentrated and desalted by isopropanol precipitation and dissolved in 30 μl TE (10 mM Tris HCl pH 8.0, 1 mM EDTA). Contaminating DNA was digested by DNAse (Promega, Madison, WI, USA), and high yields of full-length, double-stranded cDNA were then synthesised using ‘switching mechanism at 5′ end of RNA template’ (SMART, Clontech, Palo Alto, CA, USA) as previously described (Endege *et al*, 1999). Briefly, cDNA first strands were synthesized in 10 l solution containing 3 μl RNA sample, 1 μM cDNA synthesis primer, 1 μM SMART II oligonucleotide and 2 μl 5× first strand buffer (Clontech), 200 units MMLV reverse transcriptase (Gibco, Gaithersburgh, MD, USA), 1 μl dNTP mix (2 mM final concentration each) and 2 mM DDT at 42°C for 1 h. Multiple second cDNA strands were then synthesized in 100 μl volume containing 10 μl first strand cDNA, 2 μM PCR primer, 2 μl Advantage cDNA polmyerase mix and 10 μl 10× PCR buffer (Clontech) and 1 μl dNTP mix (2 mM final concentration each). Cycle conditions were: 95°C 1 min, followed by multiple cycles at 95°C 5 s, 65°C 5 s, 68°C 6 min. After 15, 18, 21 and 24 cycles, 5 μl aliquots of each PCR tube were electrophoresed on a 1.2% agarose/ethidiumbromid gel to determine the optimal number of PCR cycles with high cDNA yields but not high-molecular-weight smear. Presence of cDNA was then checked by PCR amplification of β-actin in a final volume of 50 μl containing 2.5 units Taq DNA polymerase and 5 μl 10× PCR buffer (Gibco), 0.5 μl of each primer (100 pmol μl^−1^) and 3 μl MgCl_2_ 1.5 mM. PCR conditions were: 95°C 10 min, 30 cycles of 95°C 30 s, 55°C 30 s, 72°C 30 s, followed by 72°C 10 min. Primer sequences were: SMART II oligonucleotide 5′-AAg CAg Tgg TAA CAA CGC AgA gTA CgC ggg-3′; cDNA synthesis primer 5′-AAg CAg Tgg TAA CAA CgC AgA gTA CT-3′; PCR primer 5′-AAg CAg Tgg TAA CAA CgC AgA gT-3′; β-actin-509-for 5′-TgA Cgg ggT CAC CCA CAC TgT gCC CAT CTA-3′; β-actin-1169-rev 5′-CTA gAA gCA TTT gCg gTg gAC gAT ggA ggg-3′ (human β-actin gene, accession number emhum4 : HSAC07 X00351, Ponte *et al*, 1984). All PCR and hybridization steps were performed on a GeneAmp 2400 thermal cycler (Perkin Elmer, Norwalk, CT, USA).

### Semi-quantitative reverse transcription (RT)–PCR

To compare the expression of the *PTEN/MMAC1* gene in melanomas with those in acquired melanocytic nevi, we performed semi-quantitative RT–PCRs. *PTEN/MMAC1* cDNAs were amplified using the primers PTEN-1545-for and PTEN-2277-rev in a first and the primers PTEN-1731-for and PTEN-2277-rev in a semi-nested PCR. One hundred ng of each cDNA sample were amplified in 50 μl PCRs as described above using the conditions 94°C 1 min and 18 cycles of 94°C 30 s, 60°C 30 s, 72°C 60 s in a first, and 12–24 cycles in the semi-nested reaction, followed by 72°C 10 min. Five μl aliquots of each reaction after 30 cycles were electrophoresed on 2% agarose/ethidiumbromide gels. The number of PCR cycles to detect *PTEN/MMAC1* cDNAs in melanomas was compared with those in acquired melanocytic nevi. To ensure the presence of comparable amounts of amplifyable cDNA in the PCRs, a 661 base pairs β-actin fragment was amplified as housekeeping gene in separate reactions. PCR conditions for β-actin amplification were: 95°C 1 min, 26 cycles of 95°C 30 s, 55°C 30 s, 72°C 30 s, followed by 72°C 10 min. Primer sequences were: PTEN-1545-for 5′-Agg gAg TAA CTA TTC CCA gTC-3′; PTEN-1731-for 5′-CgA Cgg gAA gAC Aag TTC AT-3′, PTEN-2277-rev 5′-Tgg TgT TTT ATC CCT CTT gA-3′ (PTEN cDNA sequence, accession number U92436; Steck *et al*, 1997). Results of the semi-quantitative RT–PCRs were statistically analysed by the logrank test for time-to-event data (Mantel-Haenszel test, Mantel and Haenszel, 1959) defining the resulting two-sided *P*-values to be statistically significant for *P*<0.05.

### Amplification and sequencing of PTEN/MMAC1 cDNAs

To screen for mutations and deletions in the coding region of the *PTEN/MMAC1* gene, we used unique primers and direct sequencing of the PCR product as previously described (Liu and Kagan, 1999). A 1475 bp DNA fragment was first amplified by PCR using the primers PP1u and PP1d (Wang *et al*, 2000). In nested PCRs, 602 and 752 bp cDNA fragments were synthesised using the primer pairs F1/R1 and F2/R2, respectively. Cycle conditions were: 94°C 1 min, 40 cycles of 94°C 90 s, 56°C 120 s, 72°C 150 s, followed by 72°C 10 min in the first PCR and 94°C 1 min, 40 cycles of 94°C 60 s, 50°C 120 s, 72°C 150 s, followed by 72°C 10 min in the nested PCRs. In each case, as a control for a successful RT of the RNA, we amplified a 661 bp β*-actin* cDNA fragment. Primer sequences were: PP1u 5′-AgA gCC ATT TCC ATC CCT gCA-3′ (nucleotides 945–964); PP1d 5′-gTg TCA AAA CCC TgT ggA Tg-3′ (nucleotides 2420–2401; Wang *et al*, 2000); F1-for 5′-AgC TTC TgC CAT CTC TCT CCT CC-3′, R1-rev 5′-gAT TCT TTA ACA ggT AgC TA-3′, F2-for 5′-Agg gAg TAA CTA TTC CCA gTC-3′ (identical with PTEN-1525-for), R2-rev 5′-Tgg TgT TTT ATC CCT CTT gA-3′ (Liu and Kagan, 1999). The DNA bands were visualized on 2% agarose/ethidiumbromide gels. *PTEN/MMAC1* amplificons were then purified and the QIAquick PCR purification kit according to the manufacturer's instructions (QIAGEN) and sequenced at a concentration of 50 ng μl^−1^ with a GeneAmp PCR system 9600 using ABI Prism dGTP BigDye Terminator Ready Reaction Kits and the AmpliTaq DNA polymerase FS according to the manufacturer's protocol (Perkin Elmer, Seqlab, Göttingen, Germany). For sequencing *PTEN/MMAC1* cDNA transcripts from both sides, the primers F-1, R-1, F-2 and R-2 were used. PCRs consisted of 25 cycles including a denaturation step at 96°C for 10 s, a primer annealing step at 50°C for 5 s and a chain elongation step at 60°C for 60 s. Cycle sequencing products were then ethanol precipitated, run on a 4% polyacrylamide 7 M urea gel and analysed with the ABI Prism 377 Genetic Analyzer (Perkin-Elmer; Seqlab). The resulting sequences were aligned to known DNA sequences in the database of the National Center for Biotechnology Information (NCBI, Bethesda, MD, USA, http://www.ncbi.nlm.nih.gov/BLAST). PTEN/MMAC1 protein sequences predicted by the cDNA sequences were compared with the PTEN/MMAC1 wild type protein sequence using the BLASTX sofware at the NCBI.

### Immunohistochemical staining of PTEN/MMAC1 protein

Serial 4 m-thin paraffin sections from formalin-fixed and paraffin-embedded melanoma and acquired melanocytic nevus tissues were deparaffinised and rehydrated in graded ethanol solutions. After retrieval pretreatment by 750 W microwave at 85°C in 0.1 M sodium citrate buffer pH 6.0 for 20 min, unspecific binding was blocked by 10% goat serum (Vector, Wertheim-Bettingen, Germany). Following overnight incubation at room temperature with the primary antibodies, the sections were washed repeatedly in destilled water and phosphate-buffered saline (PBS). Next, slides were incubated with sheep-anti-mouse-antibody as secondary antibody (Amersham-Buchler, Braunschweig, Germany) and developed using the streptavidin-biotin-peroxidase complex technique as described (Hsu *et al*, 1981). The visualisation was performed with a nickel-enhanced diaminobenzidine (DAB) reaction, resulting in a black staining of structures containing the epitope (Hsu and Soban, 1982). Sections were counterstained with hematoxylin and mounted with Eukitt® (Merck, Darmstadt, Germany). The monoclonal antibodies NCL-PTEN (Novocastra, Newcastle upon Tyne, UK, dilution 1 : 100), S100 (Linaris, Wertheim, Germany, dilution 1 : 3), HMB45 (Loxo, Dossenheim, Germany, prediluted) and CD68 (Dako, Hamburg, Germany, dilution 1 : 80) were used for the detection of the respective proteins. As a negative control, the primary antibody was substituted by equal amounts of normal mouse immunoglobulin G (Santa Cruz Biotechnology, Santa Cruz, CA, USA, dilution 1 : 100) in parallel experiments.

## RESULTS

### *PTEN/MMAC1* gene expression is not down-regulated in melanomas in comparison to acquired melanocytic nevi

Semi-quantitative RT–PCRs were done with 19 acquired melanocytic nevus and 18 melanoma tissue specimens (seven nodular melanomas, 10 superficial spreading melanomas and 1 acrolentiginous melanoma from the tumours listed in Table 1

**Table 1:**
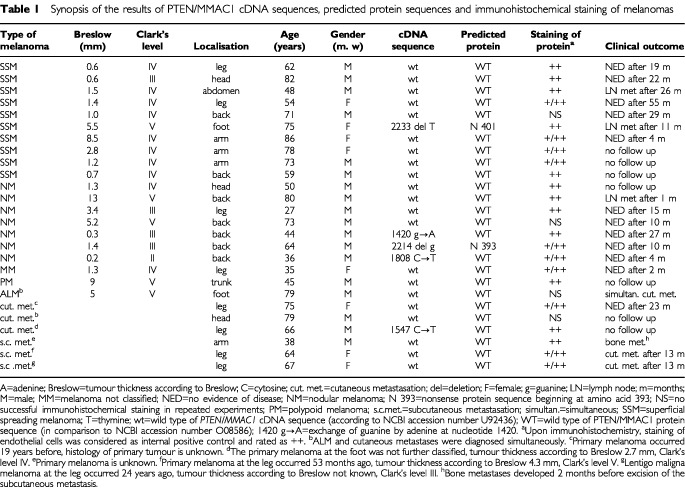
Synopsis of the results of PTEN/MMAC1 cDNA sequences, predicted protein sequences and immunohistochemical staining of melanomas

). The presence of comparable amounts of amplifyable cDNA was ensured by amplification of β-actin as housekeeping gene. Using 100 ng of each sample for 24 PCR cycles, 661 bp β-actin amplicons of comparable amounts were observed on agarose gels (data not shown). The possibility of amplification of genomic DNA was eliminated by β-actin PCRs without RT, which were negative in all specimens (data not shown). Next, expression of the *PTEN/MMAC1* gene was evaluated by semi-quantitative PCR analysis. Following 12 cycles of semi-nested PCR, a single acquired melanocytic nevus (5%) and a single melanoma (6%) were positive. After 14 cycles, five nevi (26%) and seven melanomas (39%) showed positive result. The 344 bp amplificons were seen in seven nevi (37%) and eight melanomas (44%) following 16 cycles, in 13 nevi (68%) and 14 melanomas (78%) following 18 cycles, in 15 nevi (79%) and 17 melanomas (94%) following 20 and 22 cycles. Following 24 PCR cycles, four nevi and none of the melanomas remained negative. Applying the logrank test for time-to-event data (Mantel-Haenszel test), the resulting two-sided *P*-value of 0.17 indicated no statistically significant difference of the semi-quantitative RT–PCR testing nevi and melanoma tissue samples. We have to remark that due to the relative low number of tested tissue samples the log rank test has limited power to detect differences. The estimated hazard ratio for melanomas versus nevi was 1.6 (95% confidence interval 0.81; 3.23).

### Mutations and deletions of* PTEN/MMAC1* cDNAs were found in some melanomas

**Figure 1:**
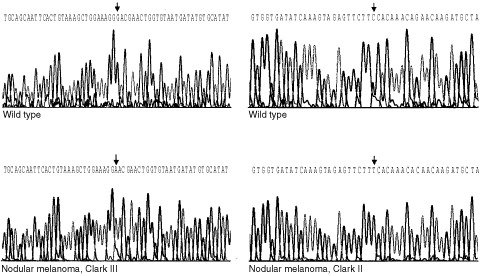
Part of the *PTEN/MMAC1* cDNA sequences in melanomas in comparison to the wild type sequences. Guanine was found to be exchanged by adenine at nucleotide 1420 in a nodular melanoma, Clark's level III (left). Cytosine was found to be exchanged by thymine at nucleotide 1808 in a nodular melanoma Clark's level II (right).

Twenty-six melanoma resection specimens (seven nodular melanomas, 10 superficial spreading melanomas, one acrolentiginous melanoma, one polypoid melanoma, one melanoma not classified, three cutaneous, three subcutaneous metastases, tumours listed in Table 1) were succesfully used for RNA purification and reverse transcription. Following PCR amplification and sequencing of *PTEN/MMAC1* cDNAs, sequences were compared with the known *PTEN/MMAC1* cDNA sequence (genbank accession number emhum7:HSU93051; Steck *et al*, 1997) using the BLASTN software. Three point mutations and two deletions were detected in five melanomas whereas wild type of nucleotide sequence was found in 21 samples (Table 1, Figure 1

).

Next, the PTEN/MMAC1 protein sequences predicted by the cDNA sequences that we found were compared with the known PTEN/MMAC1 protein sequence (PTEN_HUMAN 000633 and NM_000314, Li *et al*, 1997) using the BLASTX sofware. None of the three detected point mutations resulted in changes of amino acids or stop codons. All three point mutations were silent mutations as they did not alter the predicted protein sequence. The two deletions that we found resulted in frame shifts of the nucleotide sequence followed by subsequent nonsense protein sequence at the C-terminus of the PTEN/MMAC1 protein without protein truncation. As a consequence, the corresponding PTEN/MMAC1 proteins are predicted to have lost the normal five C-terminal amino acids, which are supposed to function as PDZ-binding consensus sequence (Leslie *et al*, 2000). Further critical motifs of the PTEN/MMAC1 protein, e.g. the catalytic domain (tyrosin phosphatase domain, Steck *et al* (1997); Ali *et al* (1999); genbank accession number O08586, PTEN/MMAC1 exon 5, amino acids 122–132) or the phosphate acceptor sites (residues 233–240, 308–315) were altered in none of the 26 predicted PTEN/MMAC1 proteins.

The presence of genetic polymorphisms was excluded by sequencing the *PTEN/MMAC1* cDNAs from corresponding blood mononuclear cells, which were regarded as non-neoplastic tissues of the patients where *PTEN/MMAC1* cDNAs were found to be altered in the melanoma.

In the clinical follow up, 19 of the 26 patients were seen in regular intervals. Progression of the disease was observed in seven patients with three cutaneous, three lymph node and one bone metastases (Table 1). Looking at the five patients with cDNA alterations, one patient with a deletion in the *PTEN/MMAC1* cDNA suffered from lymph node metastasis 11 months after primary surgery. In respect to statistical correlation of *PTEN/MMAC1* cDNA alterations with the clinical course of the disease, the number of investigated melanoma samples was too small.

### Immunohistochemical detection of the PTEN protein in melanomas and acquired melanocytic nevi

Expression of the PTEN/MMAC1 protein was analysed in six acquired melanocytic nevi, 18 primary and five metastatic melanomas. Melanoma tissues corresponded to the samples investigated for PTEN/MMAC1 gene expression and cDNA sequence. Evaluating the tissue sections of the nevi, distinct nuclear staining was seen in the majority of melanocytes which was comparable to the signals in endothelial cells with respect to intensity (data not shown). Immunoreactions were also positive in cells of the hair follicles and in part of the cells in the stratum basale of the epidermis.

**Figure 2:**
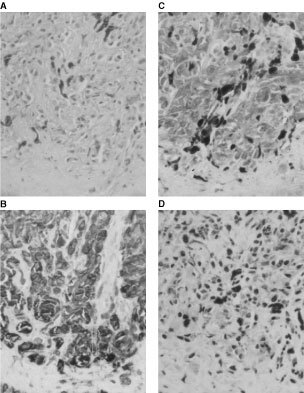
Immunhistochemical staining of serial tissue sections of a nodular melanoma, tumour thickness according to Breslow 1.4 mm, Clark's level IV. The PTEN/MMAC1 protein was detected in melanoma cells and was not restricted to tumour associated macrophages. (a) Hematoxylin Eosin, (b) HMB45, (c) CD68, (d) PTEN/MMAC1 staining (all magnification 25×, nickel enhanced DAB reaction resulting in black signals).

Nineteen of the 23 melanoma sections had accompanying vascular endothelial cells present, which showed strong PTEN/MMAC1 immunostaining in the nuclei, were graded ++, and served as internal positive control as described previously (Zhou *et al*, 2000). In these 19 melanomas, the PTEN/MMAC1 protein was detected in the melanoma cells and was not restricted to tumour associated macrophages (Table 1, Figure 2

). In most cases, the intensity of signals in the melanoma cells was comparable to that in endothelial cells and signals were located in the nuclei. In 10 melanomas, nuclear staining intensity varied from weak (+) to moderate (++) nuclear PTEN/MMAC1 immunostaining. In the remaining four of the 23 investigated melanoma tissues, immunohistochemical staining was not successful as endothelial cells remained negative as well.

PTEN/MMAC1 immunohistochemistry was also positive for those two primary melanomas which harboured a nucleotide deletion in the *PTEN/MMAC1* cDNA sequence. As these deletions at cDNA level resulted in predicted nonsense sequences of the protein at the C-terminus, the PTEN/MMAC1 protein might further be detected by the monoclonal NCL-PTEN antibody which has been produced by immunisation with the 200 amino acids at the C terminus.

## DISCUSSION

*PTEN/MMAC1* is one of the most commonly mutated genes in human cancer (Teng *et al*, 1997; Bonneau and Longy, 2000; di Cristofano and Pandolfino, 2000). As this tumour suppressor gene is located at chromosome 10q23.3 and melanomas frequently harbour deletions at chromosome 10q22-qter (Isshiki *et al*, 1993; Herbst *et al*, 1994; Walker *et al*, 1995; Healy *et al*, 1996, 1998) like carcinomas of the prostate (Trybus *et al*, 1996) and the kidney (Speicher *et al*, 1994), inactivation of *PTEN/MMAC1* has been supposed to occur in melanomas. Actually, deletions and mutations of *PTEN/MMAC1* have been found in 29–43% of investigated melanoma cell lines (Guldberg *et al*, 1997; Tsao *et al*, 1998; 2000). We therefore tested melanoma primary resection specimens for mRNA and protein expression and *PTEN/MMAC1* cDNAs for the presence of mutations or deletions.

Applying semi-quantitative RT–PCR, we could not find a statistically significant down-regulation of the *PTEN/MMAC1* expression in melanomas at mRNA level in comparison to acquired melanocytic nevi, from which melanomas are known to arise. Likewise, *PTEN/MMAC1* expression was not lost at protein level as we observed distinct nuclear signals in melanoma cells by immunohistochemistry. Our immunohistochemical data contrast to the report of Zhou *et al* (2000) who described absent or weak expression of PTEN/MMAC1 nuclear signals in four primary and 30 metastatic melanomas and assumed epigenetic *PTEN/MMAC1* silencing. Applying the same immunohistochemical protocols, the use of different primary antibodies may cause these divergent results. We used the monoclonal antibody NCL-PTEN which is commercially available while Zhou *et al* (2000) and coworkers developed their own PTEN/MMAC1-specific antibody 6H2.1.

Alterations of the *PTEN/MMAC1* cDNA sequence were detected in five of 26 investigated melanoma resection specimens, among them three silent point mutations. The two deletions that we identified are predicted to result in nonsense protein sequences at the extreme C-terminus of the PTEN/MMAC1 protein. Considering the last three amino acids as consensus sequence for binding PDZ domain containing proteins (Leslie *et al*, 2000), localisation and function of PTEN/MMAC1 are possibly altered in the corresponding two melanoma primary tumours. As PDZ domains are known to recruit components of lipid signalling pathways to particular membranes (Montell, 1998; Nemoto and de Camilli, 1999), deletion of the PDZ domain may disrupt targeting of the PTENMMAC1 protein to the plasma membrane (Leslie *et al*, 2000). Refering to the function of PTEN/MMAC1 missing the putative PDZ-targeting sequence, a PTEN/MMAC1 enzyme with a deletion of the C-terminal five amino acids (Q399STOP mutation) has been reported to retain full phosphatase activity but to be impaired in inhibiting cell spreading and platelet derived growth factor (PDGF)-induced membrane ruffling (Leslie *et al*, 2000; 2001) as well as in inhibiting colony formation (Morimoto *et al*, 1999). Similar to our findings, C-terminal frame shifts and missense mutations of PTEN/MMAC1 have previously been identified in glioblastomas (Ali *et al*, 1999; Bonneau and Longy, 2000).

The frequency of somatic mutations and deletions of *PTEN/MMAC1* in melanoma resection specimens is controversially discussed so far: Microsatellite analyses demonstrated LOH at the *PTEN/MMAC1* locus in none of 23 primary cutaneous and 17 metastatic melanomas (0%; Böni *et al*, 1998), in a single of 10 investigated melanomas (10%; Steck *et al*, 1997), in two of 44 informative primary and metastatic melanomas (4%; Herbst *et al*, 1999) and in three of eight primary and in 18 of 31 metastatic melanomas (3 and 58%, respectively; Birck *et al*, 2000). Applying denaturing gel electrophoresis, none of three primary melanomas with LOH but four of 61 melanoma metastases harboured mutations of the *PTEN/MMAC1* gene (Birck *et al*, 2000).

Reifenberger *et al* (2000) reported *PTEN/MMAC1* mutations in four of 37 investigated metastases (11%) from which two had lost one *PTEN/MMAC1* allele. Melanoma metastases seem to be affected by LOH at the *PTEN/MMAC1* locus more frequently as was further reported for 33% of 21 cases; 19% of these specimens harboured mutations in the remaining allele (Celebi *et al*, 2000). In addition, Poetsch *et al* (2001) detected nine intronic mutations in 25 primary and 25 metastatic melanoma resection specimens among them two mutations in metastases resulted in amino acid changes. If these genetic alterations are the cause or the result of metastazation, remains unclear. Mutations of *PTEN/MMAC1* in cell lines may be acquired during cell culture at least in some cases as was demonstrated for six melanoma cell lines where none of the mutations found in the cell lines have been recovered in the melanoma resection specimens from which the cell lines had been derived from (Guldberg *et al*, 1997).

Altogether, involvement of the *PTEN/MMAC1* tumour suppressor gene in melanomas remains controversial. Our data do not reveal a statistically significant down-regulation of the *PTEN/MMAC1* expression at mRNA or protein level. Among 26 melanoma resection specimens, we here describe three silent point mutations and two frame shifts of the PTEN/MMAC1 cDNA sequence. The N-terminal phosphatase domain or the phosphate acceptor sites were not affected by any mutation or deletion. The two frame shifts at the extreme C-terminus were harboured by melanoma primary tumours and resulted in a predicted loss of the putative PDZ-targeting consensus sequence probably important in localisation and function of the PTEN/MMAC1 protein. We here first report on single melanoma primary tumours that exhibit PTEN/MMAC1 proteins predicted to lack the C-terminal PDZ-binding motif, possibly impairing localization and function of this tumour suppressor protein.
